# Intracellular thermometry with fluorescent sensors for thermal biology

**DOI:** 10.1007/s00424-018-2113-4

**Published:** 2018-02-04

**Authors:** Kohki Okabe, Reiko Sakaguchi, Beini Shi, Shigeki Kiyonaka

**Affiliations:** 1 Graduate School of Pharmaceutical Sciences, The University of Tokyo, Tokyo, 113-0033 Japan; 2 JST, PRESTO, 4-8-1 Honcho, Kawaguchi, Saitama, 332-0012 Japan; 3 World Premier International Research Initiative-Institute for Integrated Cell-Material Sciences, Kyoto University, Kyoto, 606-8501 Japan; 4 Department of Synthetic Chemistry and Biological Chemistry, Graduate School of Engineering, Kyoto University, Kyoto, 615-8510 Japan

**Keywords:** Fluorescent thermometer, Intracellular temperature, Thermometry, Thermogenesis, Fluorescent sensor

## Abstract

Temperature influences the activities of living organisms at various levels. Cells not only detect environmental temperature changes through their unique temperature-sensitive molecular machineries but also muster an appropriate response to the temperature change to maintain their inherent functions. Despite the fundamental involvement of temperature in physiological phenomena, the mechanism by which cells produce and use heat is largely unknown. Recently, fluorescent thermosensors that function as thermometers in live cells have attracted much attention in biology. These new tools, made of various temperature-sensitive molecules, have allowed for intracellular thermometry at the single-cell level. Intriguing spatiotemporal temperature variations, including organelle-specific thermogenesis, have been revealed with these fluorescent thermosensors, which suggest an intrinsic connection between temperature and cell functions. Moreover, fluorescent thermosensors have shown that intracellular temperature changes at the microscopic level are largely different from those assumed for a water environment at the macroscopic level. Thus, the employment of fluorescent thermosensors will uncover novel mechanisms of intracellular temperature-assisted physiological functions.

## Introduction

Temperature is a fundamental physical quantity that is involved in all life activities. Needless to say, physiological functions are regulated by temperature at the molecular level for live animals. Temperature governs biochemical reactions in molecules as it defines their state and dynamics. At the cellular level, for instance, heat shock responses serve as a system to adapt to temperature changes of outer environments [40, 78]. To regulate the body temperature in live animals, thermogenesis is critical. It is widely accepted that rodents have brown adipose tissues (BATs) that produce heat to maintain the body temperature [7]. Recently, PET imaging has enabled visualization that this tissue also exists in adult humans [51]. Medical studies have revealed an enhanced heat production in cancers [33, 49], localized infections [64, 83], febrile seizures [14], and malignant hyperthermia [61]. Despite the fundamental involvement of temperature in life systems, little is known about the molecular mechanism regarding the expression of temperature-related physiological functions. It is easily imagined that spontaneous or adaptive thermogenesis work as the heat source for these functions, although we are still not able to answer even this simple question.

Cells possess various systems to detect environmental temperature changes. Adaptive alteration of gene expression through heat shock response and stress granule formation has been classically known [21, 59]. Moreover, several kinds of ion channels, including transient receptor potential (TRP) channels, are activated in a temperature-dependent manner [48, 80]. These functions propagate information regarding a temperature change in outer environments or plasma membranes into the interior of cells as electrical signals. Interestingly, a part of the TRP channels are expressed deep inside of the brain, which suggests that these channels are capable of sensing the changes in temperature inside of brain tissues. In *drosophila*, temperature-regulated behavior is closely linked to the rates of mitochondrial oxidative metabolism in cells [69]. These facts may reflect the existence of a temperature variation at the cellular level inside animal bodies which participates in physiological functions.

**Fig. 1:**
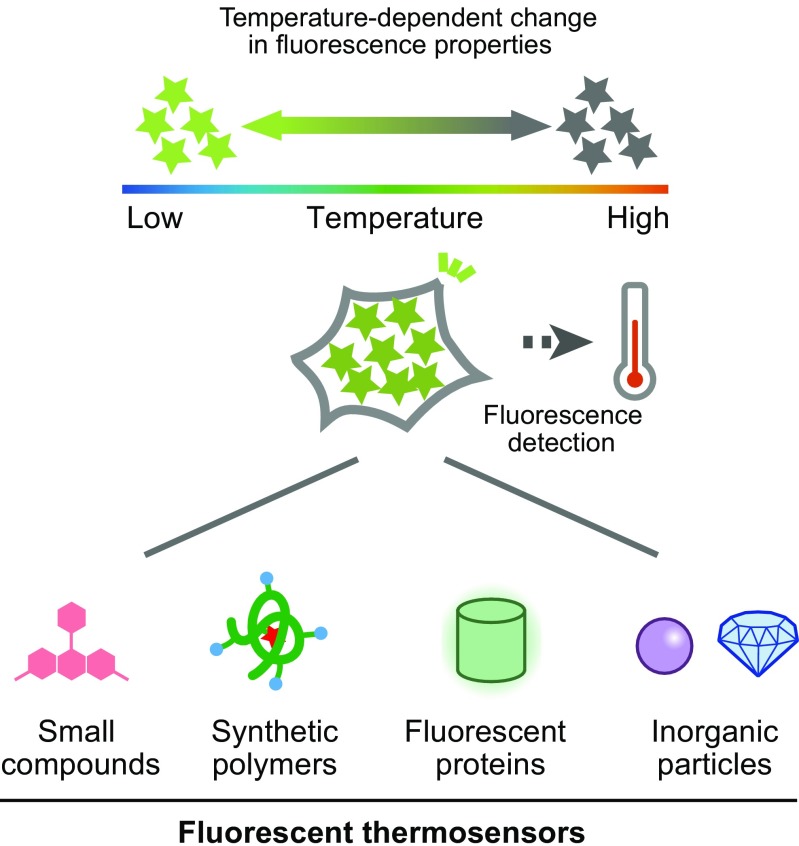
Schematic drawing of intracellular thermometry with fluorescent thermosensors

To shed light on this unexplored field, new tools to visualize intracellular thermal changes in live cells or animals are highly desired. Among the possible approaches, the fluorescence measurement is a powerful method to visualize various intracellular events. Actually, many kinds of intracellular fluorescent thermosensors have been recently developed, some of which are able to visualize subcellular thermal changes (Fig. 1 and Table 1). In this review article, we describe the fluorescent thermometers that have enabled intracellular thermometry, and how they have contributed to the observation of organelle-related heat production.

**Table 1:** Fluorescent thermosensors used for intracellular thermometry

Section	Year	Technique						Application			Ref.
		Material	Sensor name	Detection	Introduction method	Resolution (°C)	Sensitivity (%/°C)	Cell type	Localization	Observed thermogenesis	
Small organic compounds	1998	Metal complex	Eu-TTA	Phosphorescence intensity	Liposome	N/A	N/A	CHO	Cell membrane	Endogenous	[87]
	2007	Metal complex	Eu-TTA within micropipette	Fluorescence intensity	Physical contact of micropipette	N/A	2.7	HeLa	N/A	Chemically-induced	[66]
	2014	Metal complex	Eu-TTA, rhodamine 101	Fluorescence intensity ratio	Internalization	0.5	N/A	HeLa	N/A	Chemically-induced	[68]
	2014	Organic compound	ER thermoyellow	Fluorescence intensity	Internalization	0.4	3.9	HeLa, differentiated myoblast, NIH3T3, Chang, brown adipose	ER	Chemically-induced	[2]
	2015	Organic compound	Mito-RTP	Fluorescence intensity ratio	Internalization	0.6	2.7	HeLa	Mitochondria	Chemically-induced	[25]
	2015	Organic compound	Mito thermoyellow	Fluorescence intensity	Internalization	0.3	2.0–2.8	HeLa, differentiated myoblast, mESC, NIH3T3, Chang, brown adipose	Mitochondria	Chemically-induced	[3]
Synthetic polymer	2009	Polyacrylamide polymer	NIPAM-DBD (nanogel, FNT)	Fluorescence intensity	Microinjection	0.29–0.50	N/A	COS7	Cytoplasm	Chemically-induced	[20]
	2011	Polymeric micelle	ICG/Pluronic F-127/PEI micelle	Fluorescence intensity	Internalization	N/A	N/A	A431	N/A	N/A	[9]
	2011	Polymer dots (Pdots)	Pdot-RhB	Fluorescence intensity ratio	Internalization	N/A	N/A	HeLa	N/A	N/A	[86]
	2011	Polymeric micelle	HMA/Pluronic P85/TRITC micelle	Fluorescence intensity ratio	Internalization	N/A	N/A	HeLa	Cytoplasm	N/A	[16]
	2012	Polyacrylamide polymer	PNIPAm-MAn-AMC	Fluorescence intensity	Internalization	N/A	N/A	MDCK	Cytoplasm	N/A	[56]
	2012	Polyacrylamide polymer	NNPAM-DBD (FPT)	Fluorescence lifetime/Fluorescence intensity	Microinjection	0.18–0.58	N/A	COS7, HeLa	N/A	Endogenous and chemically-induced	[52]
	2013	Polyacrylamide polymer	Cationic NNPAM-DBD	Fluorescence lifetime	Internalization	0.09–0.78	N/A	Yeast, MOLT-4, HEK293T	N/A	N/A	[74]
	2014	Polyacrylamide polymer	PNIPAm/NBD/RhBAM	Fluorescence intensity ratio	Internalization	0.3–0.5	N/A	HeLa	N/A	Chemically-induced	[57]
	2015	Polyacrylamide polymer	PNIPAm/NBD/NSVB/TfAuNCs	Fluorescence intensity ratio	Internalization	0.3–0.5	N/A	HeLa	N/A	Chemically-induced	[58]
	2015	Polyacrylamide polymer	Cationic NNPAM/DBThD/BODIPY	Fluorescence intensity ratio	Internalization	0.01–0.25	N/A	MOLT-4, HEK293T	N/A	N/A	[76]
	2015	Polyacrylamide polymer	PNIPAM/CMA, PNIPAM/NBDAE, PNIPAM/RhBEA	Fluorescence intensity ratio (FRET)	Internalization	N/A	N/A	HepG2	Cytoplasm	Chemically-induced	[27]
	2015	Polyacrylamide polymer	Cationic NNPAM/DBThD	Fluorescence lifetime	Internalization	0.05–0.54	N/A	HeLa, COS7, NIH3T3	N/A	Endogenous and chemically-induced	[24]
	2017	Polyacrylamide polymer	PNIPAM/BODIPY	Fluorescence intensity/Fluorescence lifetime	Internalization	N/A	N/A	BHK	N/A	N/A	[18]
Fluorescent protein	2011	Protein and fluorescent dye system	C12FDG	Fluorescence intensity	Transformation, gene knock-in	0.7	N/A	*E. coli*	N/A	Exogenous	[45]
	2012	Fluorescent protein	GFP	Fluorescence anisotropy	Transfection	0.4	0.4	HeLa, U-87 MG	Cytoplasm	Exogenous	[12]
	2013	Fluorescent protein	GFP	Fluorescence anisotropy	Gene knock-in	0.8	1.4	*C. elegans*	Cytoplasm	Exogenous	[13]
	2013	Fluorescent protein	tsGFP	Fluorescence intensity ratio	Transfection, viral infection	0.5	N/A	HeLa, brown adipocyte, differentiated myotube	Cytoplasm, ER, mitochondria, plasma membrane	Endogenous	[35]
	2017	Fluorescent protein	gTEMP	Fluorescence intensity ratio	Transfection, gene knock-in, mRNA injection	0.4	2.6	HeLa, medaka	Cytoplasm, nucleus, mitochondria	Endogenous and chemically-induced	[50]
Inorganic materials	2010	Nano particle	NaYF_4_: Er^3+^, Yb^3+^ NPs	Fluorescence intensity ratio	Internalization	N/A	N/A	HeLa	N/A	Exogenous	[79]
	2010	Quantum dot	CdSe-QD	Fluorescence wavelength shift	Internalization	N/A	0.025	HeLa	N/A	Exogenous	[42]
	2011	Quantum dot	Quantum dot (QD655)	Photoluminescence spectral shifts	Endocytosis	N/A	0.016	HeLa, NIH3T3	N/A	Chemically-induced	[84]
	2012	Quantum dot	Quantum dot-quantum rods (QD-QRs)	Photoluminescence ratio	Cationic polymer-based incorporation	0.1	2.4	HeLa, NIH3T3	N/A	Exogenous	[1]
	2013	Quantum dot	CdSe-QD	Photoluminescence spectral shifts	Internalization	N/A	0.016	lymphocytes	N/A	Exogenous	[22]
	2013	Quantum dot	CdSe-QD	Fluorescence spectral shift	Internalization	N/A	0.025	HeLa	N/A	Exogenous	[43]
	2013	Gold nanocluster	Au nanocluster	Fluorescence lifetime	Internalization	0.3–0.5	N/A	HeLa	N/A	Exogenous	[63]
	2013	Nanodiamond	Nanodiamonds nitrogen-vacancy center	Normalized fluorescence	Nanowire-assisted delivery	0.044	N/A	Fibroblast WS1	N/A	Exogenous	[38]
	2017	Nanodiamond	Gold nanorod-fluorescent nanodiamond hybrid	Normalized fluorescence	Internalization	N/A	N/A	HEK293T	N/A	Exogenous	[72]
	2014	Silica nanoparticle	Ru(bpy)_3_^2+^ doped silica nanoparticle	Luminescence intensity	Internalization	N/A	1.26	HepG2	N/A	Exogenous	[85]
	2015	Metal nanocluster	Cu nanocluster	Photoluminescence intensity	Internalization	N/A	N/A	MC3T3-E1	N/A	Exogenous	[82]
	2015	Quantum dot	Polymer encapsulated quantum dot	Photoluminescence	Internalization	0.43	1.6	HepG2	N/A	Exogenous	[41]
	2016	Quantum dot	Quantum dot (QD655)	Fluorescence intensity ratio	Endocytosis	0.098	6.2	SH-SY5Y	N/A	Chemically-induced	[70]
	2016	Fluorescent up-converting particle	NaYF_4_:Er^3+^, Yb^3+^ nanoparticle	Fluorescence intensity ratio	N/A (Cell surrounding)	N/A	1.6	HeLa	N/A	Exogenous	[60]
Others	1998	Infrared thermography	Infrared camera	Thermal radiation	N/A (Remote monitoring)	–	–	Yeast, adipocyte	N/A	Endogenous and chemically-induced	[54]
	2011	Thermocouple	Pt-W thermocouple	Thermoelectricity	Insertion	–	–	U251	N/A	Chemically-induced	[81]
	2012	Resonant thermal sensor	Si resonator	Resonant frequency of the resonator	Physical contact	–	–	Brown fat cell	N/A	Endogenous	[30]
	2013	Photoacoustic imaging contrast reagent	Iron oxide micro-particle	Photoacoustic thermometry	Internalization	–	–	HeLa	N/A	Exogenous	[17]
	2014	Microcantilever	Bimaterial microcantilever	Microcantilever displacement	Physical contact	–	–	Brown adipocyte	N/A	Endogenous	[62]

## Fluorescent thermometers

### Fluorescent small organic compounds

#### Eu-TTA

**Fig. 2:**
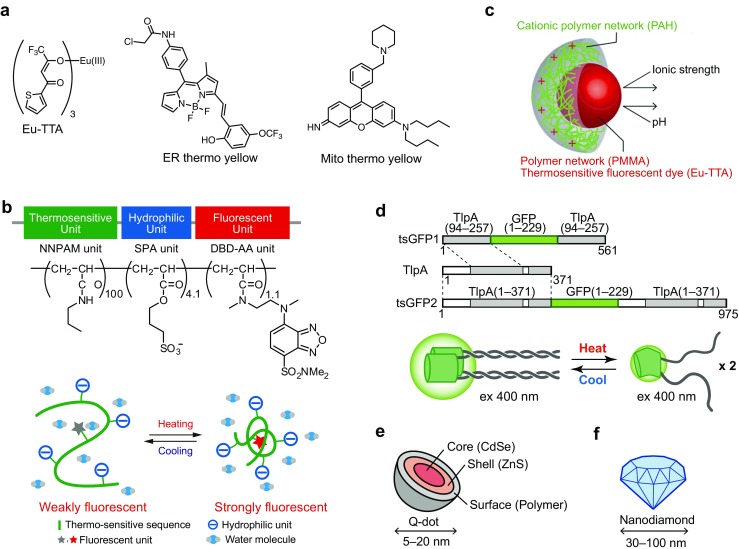
Schematic representation of fluorescent thermosensors for live single or subcellular cell thermometry. **a** Chemical structures of Eu-TTA, ER thermo yellow and Mito thermo yellow. **b** Schematic drawing of FPT. *Upper*; chemical structure of FPT. NNPAM, poly-*N*-*n*-propylacrylamide. SPA, 3-sulfopropyl acrylate. DBA-AA, *N*-{2-[(7-*N*,*N*-dimethylaminosulfonyl)-2,1,3-benzoxadiazol-4-yl](methyl)amino}ethyl-*N-*methylacrylamide. *Lower*; functional diagram of FPT in an aqueous medium. This figure is reproduced from Fig. 1 in [52]. **c** Schematic drawing of dye-embedded synthetic polymer nanothermometer. This figure is reproduced from Fig. 1 in [53] with permission. Copyright 2012, the Royal Society of Chemistry. **d** Schematic drawing of tsGFP. *Upper*; Design of tsGFPs. The gray bar indicates coiled-coil regions of TlpA. *Lower*; a schematic representation of the tandem formation of coiled-coil structure and temperature dependent fluorescence changes of tsGFP. This figure is reproduced from Fig. 1 in [35]. **e, f** Schematic drawing of quantum dots in **e** and fluorescent nanodiamonds in **f**

One of the earliest reports on the visualization of intracellular thermogenesis was accomplished by a thermosensitive fluorescent dye, Europium (III) thenoyltrifluoroacetonate trihydrate (Eu-TTA) (Fig. 2a) [87]. The fluorescence intensity of Eu-TTA decreases in response to an increase of the temperature, which makes it possible to monitor the temperature change in a CHO cell membrane. This probe successfully imaged the intracellular heat waves evoked by the metabotropic m1-muscarinic receptor stimulation. However, this technique is substantially affected by pH, which is a limitation for monitoring bona fide intracellular temperature change.

To overcome this drawback, Suzuki et al. developed a microthermometer consisting of a glass micropipette filled with Eu-TTA [66]. Using this thermometer, they demonstrated a time delay in the heat production of a single HeLa cell directly in contact with the glass micropipette, following ionomycin-induced Ca^2+^ influx from the extracellular space. This time delay was inversely proportional to the extracellular Ca^2+^ concentration, and the increase in temperature was suppressed when the activity of Ca^2+^-ATPases was blocked. However, this technique only monitored the temperature from outside of the cellular membrane.

#### Organelle-targeted small molecules

Recently, small-molecule fluorescent thermometers that target an intracellular organelle have been developed. To enable visualization of the temperature in the ER, a small organic molecule fluorescent thermometer, termed ER thermo yellow, has been reported (Fig. 2a) [2]. ER thermo yellow has a high sensitivity (3.9%/°C) and stains the target organelle evenly, which enables the monitoring of intracellular temperature gradients generated by external heat sources in various cell types.

A small-molecule fluorescent thermometer targeting mitochondria has been developed by several groups. Arai et al. reported Mito thermo yellow, which successfully monitors the mitochondrial temperature gradient generated by exogenous heating in various cells (Fig. 2a) [3]. A mitochondria-targeting ratiometric temperature probe, Mito-RTP has been reported by Homma et al. [25]. The fluorescence intensity of Rhodamine B decreases linearly with elevated temperature, while the fluorescence intensity of the CS NIR dye is stable at various temperatures. In the Mito-RTP, these two fluorophores are coupled at each end of the linker to provide a ratiometric property that is suitable for the selective determination of the mitochondrial temperature.

These recent studies indicate that small chemical probes have the potential to monitor the temperature at the intracellular organelle level.

### Synthetic polymer-based thermosensors

#### Polyacrylamides: thermo-sensitive polymers

Polyacrylamides, such as poly-*N-*Isopropylacrylamide (NIPAM) or poly-*N*-*n*-proplylacrylamide (NNPAM), are well-known for their structural changes in response to changes in temperature [55], which is why they have opened a door to the development of molecular thermometers. A unique characteristic of the temperature sensitivity of polyacrylamides is the phase transition, which enables a sharp response to a temperature change. Furthermore, the range of temperature can be adjustable by synthesizing a block copolymer containing more than one polymer having a different temperature sensitivity. For example, the response of fluorescent polymeric thermometers having various kinds and ratios of acrylamide derivative (*N*-*n*-propylacrylamide, *N*-isopropylacrylamide, and/or *N*-isopropylmethacrylamide) differed from one another in their sensitive temperature ranges between 20 and 49 °C [75]. The modifications that can improve the sensor (thermosensing) properties include an addition of hydrophilic unit and/or fluorescence unit; however, the incorporation of too much of these will deteriorate the response to temperature change. Uchiyama S et al. have developed highly sensitive fluorescent polymeric thermometers (FPTs) by combining NIPAM and NNPAM with a water-sensitive fluorophore, such as benzofurazans [77]. We have established FPTs that can be applied to living cells through an addition of a hydrophilic unit and the optimization of the unit composition (i.e., thermosensitive, fluorescent and hydrophilic units) (Fig. 2b). In 2009, the first demonstration of intracellular thermometry was performed using a fluorescent nanogel thermometer (FNT) that contained a cross-linker unit [20]. The nanometer-sized gelation of the FNT minimalized the interaction of the fluorescent unit with cellular components and maximized the fluorescent response to the temperature-dependent structural change. By measuring the total fluorescence intensity of the FNT microinjected into COS7 cells, we have demonstrated tracking the average temperature inside of single living cells. FNT-based intracellular thermometry on apoptotic cells and mitochondrial uncoupler-treated cells showed a significant intracellular temperature increase (0.3–1.8 °C for 2 h in apoptosis and 0.45 °C for 30 min after uncoupling), which uncovered the existence of a temperature variation inside of single living cells for the first time. In contrast to the prominent sensitivity and ability to track the temperature by using FNT, the dot-like distribution of intracellular FNT hinders the measurement of the temperature distribution in single cells. This challenge motivated us to develop a novel FPT that evenly distributes inside of cells. We then applied FPT to fluorescence-lifetime imaging microscopy (FLIM) to visualize the temperature distribution inside single living COS7 cells, which revealed the inhomogeneous temperature distribution in single steady-state cells [52]. In particular, the intracellular temperature gradient relating organelles, such as the nucleus, mitochondria (in part), and centrosome, have attracted significant attention in biology because these results strongly suggest the intrinsic relation of the intracellular temperature with cell functions. Furthermore, Uchiyama and Qiao have introduced a novel functional unit to the acrylamide-based thermometer to improve the sensor properties [24, 57, 58, 76]. For example, the introduction of a cation unit realized cell-permeable FPT that does not require microinjection into cells [24], and two kinds of fluorescence units enable ratiometric detection of the temperature [57, 76].

#### Temperature-sensitive dye-embedded synthetic polymers

As described in “Fluorescent small organic compounds” section, the weakness of Eu-TTA was the pH sensitivity. To overcome this drawback, Oyama et al. designed fluorescent nanoparticles, termed “nanothermometers,” in which Eu-TTA as a temperature-sensitive dye was embedded in a poly(methyl methacrylate) (PMMA) network (Fig. 2c) [53]. They employed the polymer network to protect the dye from the changes in pH and ionic strength. The nanothermometers spontaneously entered living HeLa cells, and visualized the temperature rise induced by laser irradiation. This was further improved with the introduction of an Eu-TTA-based ratiometric nanothermometer (RNT) [68]. Both the Eu-TTA, as the thermo-sensitive fluorophore, and Rhodamine 101, as a reference, were embedded in a polymeric particle to protect the fluorophores from intracellular conditions. The ratiometric measurement at single RNT spots was independent of the displacement of the RNT along the *Z*-axis. Therefore, the temperature could be determined at the location of each RNT under an optical microscope regardless of the dynamic movement of the living cells. As a demonstration of the spot-by-spot intracellular thermometry, the temperature changes at individual RNT spots in a single cell were monitored, where the Ca^2+^ increase was induced by the Ca^2+^ ionophore. The temperature increase was different for different spots, which suggested a heterogeneous heat production in the cell.

These diverse approaches of thermal detection using fluorescent polymers contribute to the detection of localized themogenenesis regarding the nucleus [52] and vesicle trafficking [53], and the thermometry in various cell species such as brown adipose tissue cells [23] and yeast cells [74] are described in the “Intracellular temperature dynamics” section in more detail.

### Fluorescent protein-based thermometers

Engineered fluorescent proteins are also used for monitoring the intracellular temperature. One example is the use of the green fluorescent protein (GFP). Donner et al. reported the use of GFP as a thermal nanoprobe suited for intracellular temperature mapping by monitoring the fluorescence polarization anisotropy [12]. This method was tested in HeLa or U-87 MG cells transfected with GFP by monitoring the fluorescence response to heat generated by photo-thermal heating of gold nanorods surrounding the cells. Furthermore, they also demonstrated intracellular temperature mapping in an in vivo model using *C. elegans* stably expressing GFP in neurons by the local photoheating of gold nanoparticles [13]. Although these reports indicate the applicability of GFP as a genetically encoded thermosensor, the insufficient fluorescence changes of GFP only yielded a low signal-to-noise ratio.

As an alternative method overcoming this limitation, we have developed genetically encoded GFP-based thermosensors (thermosensing GFPs: tsGFPs) that enable visualization of thermogenesis in discrete organelles within living cells (Fig. 2d) [35]. tsGFPs consist of the fluorophore-forming region of GFP inserted between tandem repeats of the coiled-coil region of the TlpA protein, an autoregulatory repressor protein in *Salmonella* that senses temperature changes [28]. The thermosensing capability is derived from a rapid and reversible structural transition from a parallel coiled-coil dimer to two unfolded monomers at around 37 °C. The excitation peaks at 400 and 480 nm of GFP (emission: 510 nm) represent the neutral and anionic forms of the GFP chromophore [73], and the fluorescence (ex400/ex480) ratio is largely dependent on the protein structure [10]. In tsGFPs, a temperature elevation increases the magnitude of the 480 nm peak and decreases that of the 400 nm peak, which results in a sigmoidal change in the fluorescence ratio across the temperature-sensing range of TlpA. This temperature dependent fluorescence change is reversible, and the temperature-sensing range of tsGFPs can be controlled by selecting the appropriate coiled-coils of TlpA. In addition, tsGFP was fused to specific organelle-targeting sequences to express tsGFPs in the plasma membrane, endoplasmic reticulum (ER), and mitochondria.

Nakano et al. have reported a genetically encoded ratiometric fluorescent temperature indicator, gTEMP, by using two fluorescent proteins, namely Sirius and mT-Sapphire with different temperature sensitivities [50]. The function mechanism of gTEMP lies in the ratiometric detection of thermo-sensitive Sirius fluorescence (425 nm) and thermo-insensitive Sapphire fluorescence (509 nm) with an excitation of 360 nm. This strategy enabled a fast tracking of the temperature change with a time resolution of 50 ms. This method was used to observe the spatiotemporal temperature change between the cytoplasm and the nucleus in cells, and quantified thermogenesis from the mitochondrial matrix in a single living cell. Moreover, the temperature in a living medaka embryo was monitored for 15 h and showed the feasibility of in vivo thermometry in living species.

Overall, genetically encoded fluorescent thermosensors can be expressed in cells or live animals non-invasively and are explicitly targeted to defined organelles by attaching the localization signal sequences to monitor subcellular thermal changes in these organelles.

### Inorganic materials

#### Quantum dots

Quantum dots (QD), semiconductor nanoparticles that emit fluorescence, have been applied to measure the temperature in living cells (Fig. 2e [47]. The luminescence properties of QDs undergo temperature-dependent optical changes, such as a red-shift of the photoluminescence peak and decrease of the fluorescence intensity upon heating. Maestro et al. reported the use of two-photon excitation of QD to observe the sharp response of the emission intensity decrease when applying an artificial heat source in HeLa cells [42]. Yang et al. used streptavidin-coated QD of CdSe/ZnS introduced into NIH/3T3 cells to observe a change in the emission peak of 0.057 nm/°C when cells were heated from 17.3 to 47.2 °C [84]. QD-based intracellular thermometry in NIH/3 T3 cells demonstrated a 2 °C increase in response to Ca^2+^ elevation upon ionomycin treatment. More recently, the change in the fluorescence wavelength of QDs loaded in neuronal SH-SY5Y cells showed a temperature increase in chemically uncoupling mitochondria [70].

#### Nanodiamonds

Nitrogen-vacancy centers (NVCs) in nanodiamonds, a fluorescent nanoparticle with unique optical characteristics, have attracted high expectation for sensing various physical parameters (Fig. 2f). An optically detected magnetic resonance (ODMR) spectrum of nitrogen-vacancy spins in nanodiamonds changes according to the temperature, which allows measurement of the local temperature in living cells [26]. Kusco et al. introduced NVCs into a human embryonic fibroblast to measure the local temperature change, which was dependent of the distance from a gold nanoparticles-assisted artificial heat source [38]. Tsai et al. developed a nanohybrid of gold nanorod-fluorescent nanodiamond as a combined nanoheater/nanothermometer to investigate the local temperature required for hyperthermia on the membrane nanotubes in HEK293T cells and found an interesting gap in the damage threshold between local and global heating [72].

#### Inorganic nanoparticles

Some inorganic fluorescent nanoparticles are also available for intracellular thermometry. The functions of these nanothermometers depend on a unique temperature sensitivity of their luminescence properties. Lanthanide-doped fluorescent nanoparticles, composed of rare earth elements, undergo near-infrared (NIR) light excitation and emit visible light by up-conversion. The temperature dependency of this multiphoton process can be used to measure the temperature. Jaque and Sole et al. have demonstrated the intracellular thermometry by using lanthanide-doped fluorescent nanoparticles in living cells and observed a gold nanorods-activated temperature change in living HeLa cells [32]. Other nanoparticles with thermo-responsive luminescent agents, such as Ru(bpy)_3_^2+^ [85], gold nanoclusters [63], and copper clusters [82], have also been shown to function as nanothermometers in living cells.

Even though the use of these fluorescent nanoparticles has been limited to single-point measurements of the local temperature using external stimuli in single cells, the remarkable photo-stability and the low-interactivity of these thermosensors with intracellular components will deepen and expand the range of intracellular thermometric investigations.

### Other technology not relying on fluorescence

Several other techniques not relying on fluorescence as the output signal have also been developed. Although these methods are not the focus of this review, we would like to briefly introduce these technologies.

#### Infrared thermography

In an earlier study, conventional thermographic detection of mitochondria-related cellular thermogenesis was reported. By observing thermal radiation from mass of cells in a dish with infrared thermography, Paulik et al. observed thermogenesis from yeasts and adipocytes upon mitochondrial stimulation (FCCP treatment and UCP expression) with 0.002 °C sensitivity [54]. This result indicates the feasibility of measuring intracellular thermogenesis outside of cells.

#### Photoacoustic microscopy

Gao et al. reported a novel single-cell photoacoustic thermometric method for intracellular thermosensing [17]. This unique technique detects ultrasound signals induced by light absorption. By measuring the photoacoustic signal generated by iron oxide micro-particles loaded into HeLa cells, they observed the intracellular temperature during photo-thermal heating. This method offers a high sensitivity (0.2 °C) and high spatial (diffraction limited level) and temporal (3 s) resolution of the single-cell thermometry.

#### Metal thermal sensors for single cells

Inomata et al. have developed novel ultrasensitive resonant thermal sensors, which are applicable in detecting thermogenesis at single-cell level [30]. These micrometer-sized sensors possess high heat conductance, which enables the detection of a temperature change even outside of the cells. The temperature resolution of this type of thermal sensors is as small as 79 μK. The biggest advantage of this method is the ability to measure the temperature change of single cells without introducing any molecules into the cells. They also demonstrated chemically-induced thermogenesis in BAT cells using a bimaterial microcantilever and a resonant thermal sensor surrounded by vacuum in a microfluidic chip [31, 62].

## Intracellular temperature dynamics

### Mitochondria

Heat generation in living organisms is not uniform, but instead, mainly occurs in specific tissues such as BAT and skeletal muscle. Especially, brown adipocytes in BAT have been well studied, and mitochondria in brown adipocytes are known to be involved in thermogenesis [7]. Thus, thermogenesis in mitochondria has also been a focus for visualizing intracellular thermal changes not only in brown adipocytes but also in other cells.

**Fig. 3:**
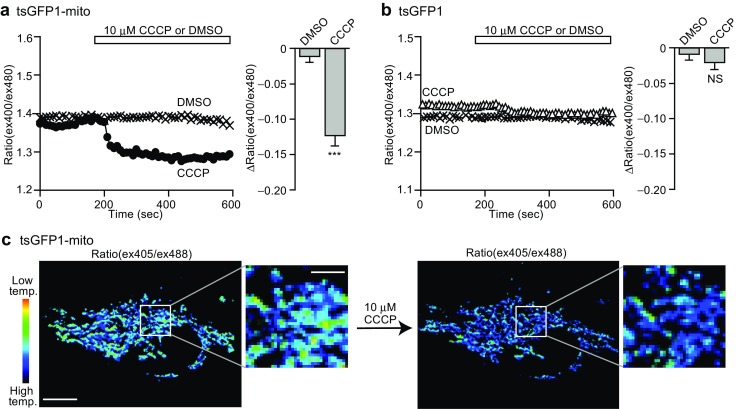
tsGFP1-mito revealed mitochondrial thermogenesis. **a**, **b** Fluorescent responses to 10 μM CCCP in HeLa cells transfected with mitochondrial tsGFP1-mito (in **a**) or cytosolic tsGFP (in **b**). *Left*; averaged time courses. *Right*; maximal ratio changes (Δratio(ex400/ex480)) after CCCP application. ***P < 0.001 by Student’s *t* test. NS not significant. **c** Pseudocolor confocal images of ratio(ex405/ex488) in tsGFP1-mito-expressing HeLa cells before and after CCCP treatment revealed thermal heterogeneity in mitochondria. Scale bars indicate 10 μm (whole image) and 3 μm (inset). This figure is reproduced from Fig. 4 in [35]

In brown adipocytes, uncoupling protein 1 (UCP1), which is selectively expressed in brown adipocytes, cancels the proton gradient of the mitochondrial membrane, resulting in heat generation instead of ATP production [15, 44]. As a model of proton gradient cancelation, chemical uncoupler CCCP (carbonyl cyanide 3-chloro-phenylhydrazone) or FCCP (carbonyl cyanide 4-(trifluoromethoxy)phenylhydrazone) are widely used in various cell lines [54, 71]. In our observations, tsGFP1-mito, the genetically encoded thermosensor specifically targeted to the mitochondria, clearly revealed the temperature increase induced by CCCP (Fig. 3a, b) [35]. We have also reported that both FNT and FPT captured the temperature rise in COS7 cells induced by FCCP, and the change in temperature was estimated to be ~ 0.45 to 1.02 °C [20, 52]. Quantum dots were also used to visualize the heat production in the mitochondria of SH-SY5Y cells by CCCP [70]. In addition, Mito thermo yellow, a mitochondria-targeted small molecular probe, was successfully demonstrated to monitor the intracellular temperature gradient generated by exogenous heating in various cells, such as HeLa, C2C12 and brown adipocytes [3].

Stimulation of a β3-adrenoreceptor, such as by norepinephrine, is known to be a physiological stimulus for activating UCP1 to induce heat generation in brown adipocytes [7]. tsGFP1-mito successfully captured norepinephrine-induced endogenous thermogenesis in brown adipocytes [35]. FPT also revealed the intracellular temperature elevation (~ 1.29 °C) in brown adipocytes upon β 3-adrenoreceptor selective agonist treatment [52]. Interestingly, an ASK1 deficiency, which leads to impaired metabolic responses including UCP1 expression in BAT and oxygen consumption of mice, resulted in restricted thermogenesis in brown adipocytes (~ 0.52 °C) [23]. This result proved the causal correlation between intracellular thermogenesis and the metabolic response of individuals.

Notably, tsGFP1-mito has revealed a heterogeneity in the distribution of the temperature among mitochondria in HeLa cells (Fig. 3c) [35]. A subpopulation of mitochondria expressing tsGFP1-mito showed a prominent increase of the CCCP-induced thermogenesis, whereas in a different subpopulation, only a subtle thermal change was detected. To further investigate the apparent thermal heterogeneity among mitochondria, tsGFP1-mito in HeLa cells was simultaneously imaged using JC-1, a dye visualizing a high mitochondrial membrane potential [65], or ATeam, a genetically encoded ATP sensor [29]. This investigation revealed that the temperature is high in the subpopulation of mitochondria where the membrane potential is high, and that the ATP level positively correlates with the membrane potential. Thus, we concluded that constitutive thermogenesis occurs through the respiratory chain or oxidative phosphorylation in a subpopulation of mitochondria in HeLa cells. To the best of our knowledge, this is one of the first examples to directly confirm the correlation between intracellular thermogenesis and organelle function.

### Endoplasmic reticulum (ER)

In addition to brown adipose tissues, skeletal muscles have been reported to be a main source of heat in many birds and animals [4]. However, whether muscles act as heat-generating organs remains controversial [8]. A mechanism has been proposed to relate thermogenesis to reactions involving ATP turnover, such as the maintenance of a Ca^2+^ gradient mediated by the sarco-endoplasmic reticulum Ca^2+^-ATPase (SERCA) pump [46], which pumps Ca^2+^ from the cytosol into the sarcoplasmic reticulum lumen using energy derived from ATP hydrolysis [11].

To directly evaluate the thermogenesis from ER in skeletal muscle myotubes, tsGFP1-ER was expressed in myotubes differentiated from myogenic C2C12 cells [35]. In tsGFP1-ER-expressing differentiated C2C12 myotubes, application of cyclopiazonic acid, a reversible inhibitor of SERCA, showed the decrease of the temperature. In contrast, this phenomenon was not observed in control C2C12 myotubes expressing GFP-ER or in undifferentiated C2C12 cells expressing tsGFP1-ER. Notably, in differentiated myotubes, the expression of SERCA1, which is the only active subtype in the fast muscles, was increased. Thus, the observation by tsGFP1-ER clearly supports the existence of thermogenesis in myotubes which is mediated by the Ca^2+^-ATPase activity of SERCA1.

An ER-targeted small-molecule thermosensor, ER thermo yellow, has demonstrated the ability to monitor intracellular temperature gradients generated by external heat sources in various cell types [2]. This sensor was tested to monitor the heat production by intracellular Ca^2+^ changes in HeLa cells and revealed the dynamics of heat production in real time at a subcellular level. Thus, these organelle-specific thermometers will be powerful tools to clarify the controversial debate regarding the thermogenesis in ER in muscles.

### Nucleus

The nucleus possesses central roles in cell functions and is uniquely characterized by the nuclear membrane of lipid bilayers, extremely complicated structure and highly active biochemical reactions including transcription.

**Fig. 4:**
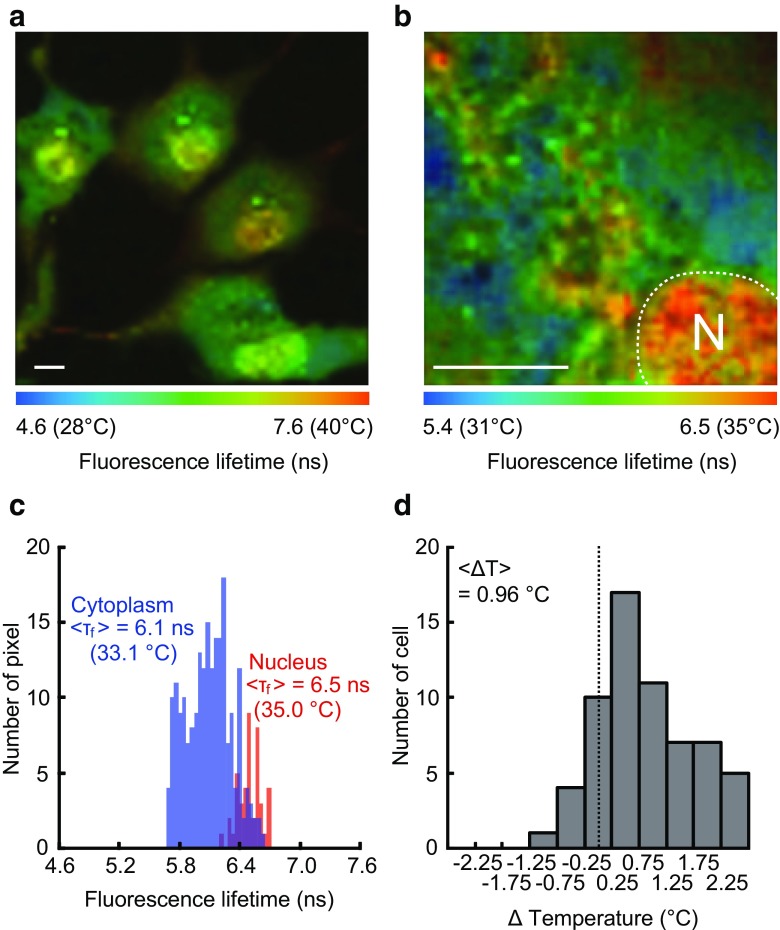
The higher temperature in the nucleus of COS7 cells observed with FPT. **a** Fluorescence lifetime image of FPT. Scale bar indicates 10 μm. **b** The temperature distribution in the local area of cell. N represents the location of the nucleus. Scale bar indicates 10 μm. **c** Higher temperature in the nucleus. Histograms of the fluorescence lifetime in the nucleus and in the cytoplasm in a representative cell (the leftmost cell in **a**). **d** Histogram of the temperature difference between the nucleus and the cytoplasm (*n* = 62 cells). ΔTemperature was calculated by subtracting the average temperature of the cytoplasm from that of the nucleus. <Δ*T*> represents an average of the histogram. This figure is reproduced from Figures 4 and 6 in [52]

FLIM using a polymer-based thermosensor, FPT, has revealed that the nucleus of COS7 and HeLa cells showed a higher temperature than the cytoplasm through temperature mapping inside the cells (Fig. 4) [52]. The temperature in the nucleus of the steady-state living cells turned out to be significantly higher than that in the cytoplasm with an average difference of 0.96 °C. Interestingly, this temperature gap between the nucleus and the cytoplasm was dependent on the cell cycle: the nucleus shows a higher temperature in the G1 phase just after cell division, while this temperature difference disappears in the S/G2 phase just before cell division. This phenomenon can be explained by the variation of the cytoplasmic temperature. In fact, an earlier study on the calorimetric detection from the mass of cells showed a G2-phase specific intensive thermogenesis. In the G2 phase, the metabolic activity of cells increases because cells must prepare the cellular mass, such as lipids and proteins, ahead of cell division. In contrast, the temperature in the nucleus remains relatively constant.

The nuclear temperature was also investigated by using the nucleus-targeted fluorescent protein thermometer gTEMP [50]. The fluorescence ratio obtained in the steady-state HeLa cell nucleus was significantly lower than that in the cytoplasm, which suggested that the nucleus had higher temperature by couple of degrees. The difference in the absolute value of the temperature gap between this result (2.9 °C) and that observed by FPT (0.96 °C) might originate from the different fine distribution of the thermometers and/or an error in the calibration, which calls for a detailed assessment in the future. Nonetheless, the fact that two methods having totally different detection principles displayed a higher temperature in the nucleus highlighted the likeliness for the thermogenic property of this organelle.

The search for the origin of nuclear heat production has just begun. The dominant chemical reactions in the nucleus, which include transcription and RNA processing, in association with various highly organized structures may be clues to uncover the mechanism of thermodynamics in the nucleus.

### Cytoskeletons (microtubule)

It is suggested that cytoskeletons, which support the principal organelles and create intracellular structures, contribute to the intracellular thermal variations.

We have shown the temperature of a centrosome, the main microtubule organizing center, is higher than the surrounding area in COS7 and HeLa cells [52]. The cell event/reaction relating to this temperature gradient near the centrosome with considerable cell-to-cell variations has yet to be elucidated, and the cause of thermogenesis at the centrosome remains an open question. Centrosome-related biochemical reactions, such as hydrolysis of tubulin-GTP, ATP-driven motion of motor proteins, and phosphorylation/dephosphorylation of centrosomal proteins by kinase/phosphatase, may be heat sources at this organelle.

It has been revealed that microtubule-related transport is highly influenced by temperature change. The transport of cellular components along microtubules accelerates when they are heated both in solution [34] and in living HeLa cells [53]. This temperature dependency arises from the ATPase activity of motor proteins. This suggests that the activity of microtubule-bound motor proteins and maybe other microtubule-associating proteins in general is influenced by the temperature change inside of cells. Taking into consideration that the thermogenic organelles, such as mitochondria and ER, are directly associating with microtubules in cells, the thermal properties of microtubules are an excellent target for investigating the relation between intracellular heat production and cell functions. This might involve the heat capacity of microtubule-bound components and/or the use of heat in microtubule-dependent transport.

## Validation of thermal changes visualized by thermosensors

Recently, a critical issue has been raised by Baffou et al. regarding the rise of Δ*T* in temperature *T* observed in single cells, which questioned the subcellular thermal heterogeneities described above [5]. They have argued that the value of Δ*T* for the whole cell should be much smaller than the observed value (a few degrees) based on the equation:

1 $$ \Delta T=\frac{P}{\kappa L} $$

where the temperature of a continuous medium of thermal conductivity *κ* on the surface of a heat source of characteristic dimension *L* and power *P* is Δ*T* higher than that at a point infinitely far from the heat source. Equation (1) is obtained from the macroscopic heat diffusion equation for continua:

2 $$ c{\partial}_tT\left(\mathbf{r},t\right)-\kappa {\nabla}^2T\left(\mathbf{r},t\right)=p\left(\mathbf{r},t\right) $$

where *c* is the volumetric heat capacity and *p*(**r**,*t*) is the heat source density as a function of position *r* and time *t*.

Baffou et al. estimated that the temperature increase would be in the order of 10^−5^ K within a single-cell from Eq. (1), which is based on the assumption of the heat source size (10 μm) and previously reported heat production (100 pW) from a cell. Taking further into consideration that the actual heat production occurs transiently within an intracellular organelle, they have subsequently calculated that the energy required to increase the temperature 1 K over a volume of 1 μm^3^ during 1 s would be 1 μJ, which is still one or two orders of magnitude higher than the energy produced by the total glucose amount contained within a whole cell.

We noticed that the values of the parameters used in their calculation of Δ*T* need to be reconsidered: Baffou et al. assumed that the medium surrounding the heat source is a homogenous and continuous water solution and employed only a single value of *κ* = 1. As is easily imagined, however, the interior of a cell is completely different from this condition. The *κ* value for proteins, which are localized in high concentrations in cells, is reported to be 0.1–0.2 Wm^−1^ K^−1^ ([39]); and the typical size of the heat source, for example mitochondria, is less than 100 nm (for details, see Ref. [36]).

More importantly, we question the applicability of the *macroscopic* heat diffusion Eq. (2) in an intracellular space. Equation (2) assumes that the *local equilibrium* is preserved at every “point” (in a macroscopic sense) in the surrounding medium under consideration. However, the fluctuation of thermodynamic quantities is significant and violates the prerequisite of “homogeneity” in “small (< 1 μm)” systems [37]. In fact, the size of the heat sources, namely protein complexes and intracellular organelles, are only 10–100 nm, in which thermal equilibrium is not fitted. Additionally, several heat sources, as well as exothermic or endothermic reactions, exist in a discontinuous manner in cells. Therefore, the assumption that the heat diffusion equation is applicable to intracellular environments should be challenged, which is more critical when considering the calculation in the aforementioned argument. To understand the thermal dynamics inside of cells, we need to chemically assess the intracellular heat sources and their thermal interaction with intracellular molecules and to develop a theory for mesoscopic non-equilibrium thermodynamics, which are all indispensable open questions in this field.

Experimental evidence strongly supports our view presented above. A number of thermometric methods, which have distinctive temperature-responding chemistry, have shown an increase of ~ 1 °C in the intracellular temperature, which presents the most decisive evidence of this phenomena. In particular, in our observations, tsGFP1-mito or tsGFP1-ER clearly revealed the significant temperature increase, in contrast to tsGFP1 expressed throughout the cytosol [35]. This difference between tsGFP1 constructs supports the view that the generated heat was transferred directly to organelle-targeted tsGFP1s in the same compartment, but reached the cytosolic tsGFP1 only after being diffused, which indicated a non-equilibrium state of intracellular local heat in cell. This is also the case in the observation of the higher temperature of the nucleus compared with the cytosol, which was visualized using a polymer-based thermosensor, FPT, by our group [52]. These results clearly indicate that it is critical to assess the effects of different subcellular compartments through precise evaluation using thermosensors targeted specifically to the respective organelles.

As just mentioned, heat generation in living organisms is not uniform, instead it occurs in specific tissues including brown adipocytes and skeletal muscles. Experimental evidence, which was demonstrated by non-fluorescent thermosensors, completely contradict Baffou et al.’s proposal that the temperature increases of a few degrees is achieved only by the collective effects of the 10^13^–10^14^ cells comprising our whole body. To name a few, the heat produced has been visualized in single brown adipocytes using a bimaterial micro cantilever and in masses (10^5^ cells) of differentiated adipocytes using infrared thermography (IRT) [54]. These methods of thermal detection *outside* of cells revealed a ~ 1 °C higher temperature in brown adipose tissue compared with its surroundings. IRT also showed a temperature heterogeneity at sub-millimeter ranges in kidneys [19]. The fact that various detection methods, inside or outside of cells, are able to capture intracellular heat production at both the cellular and the tissue level strongly supports our conclusion that the heat sufficient to raise the intracellular temperature by a few degrees is actually being produced in cells (see also [6, 67] for detailed discussion).

## Discussion and conclusions

In this review article, we describe the fluorescent thermometers that have allowed for intracellular thermometry, and how they have contributed to the observation of organelle-related heat production. Interesting spatiotemporal variation of local temperature in single cells, revealed by imaging with fluorescent thermometers, indicates their capability for providing a new perspective of physiological phenomena through intracellular temperature variation.

It is highly beneficial for thermal biologists that various fluorescent thermosensors having distinct principles are available as it enables diversified intracellular thermometry. An important insight into the temperature-sensing mechanism can be obtained from fluorescent thermometers; the common functional principle of FPT and tsGFP that has revealed organelle- and/or cell functions-related temperature variation is the phase transition. FPT and tsGFP use the temperature-dependent phase transition of polyacrylamide and TlpA, respectively, in their sharp response to heat produced in single cells. Taking into an account that endogenous macromolecules, such as ion channels and RNAs, sense any physiological temperature change in cells, the non-linear response (i.e., the aforementioned sharp response) to a temperature change of these macromolecules are highly advantageous in perceiving the temperature change inside of cellular environments. In addition to organic polymer-based or genetically encoded fluorescent thermosensors, inorganic nanoparticles thermosensors such as fluorescent nanodiamonds, quantum dots, and up-conversion fluorescent nanoparticles, have been developed; the remarkable photo-stability and low-interactivity with intracellular components allow for long-duration monitoring, and multi-modal measurements, of intracellular temperature and thereby contribute to future biological and physiological applications. Moreover, small molecule-based fluorescent thermosensors possess the remarkable ability of cellular uptake, enabling the realization of minimally invasive and broad thermometric applications.

In the future, the applications of fluorescent thermometers will drastically expand in all fields of life science. In particular, intracellular thermometry of three-dimensional cells in vivo, and cells of various species, including poikilothermic animals, homeothermic animals and plants, will be essential in this field. Nevertheless, some barriers still remain to be overcome to achieve this progress. Intracellular thermometry in vivo (including plants) would require a genetically introducible fluorescent thermometer with longer wavelengths. An adjustment or extension of the range of temperature response will be necessary because most phase transition-assisted thermometers react to a limited range of temperatures.

In conclusion, the emerging techniques of fluorescent thermometers have just started to uncover the mysteries of physiological phenomena such as the thermal heterogeneity. Further investigation into the sensing and use of intracellular heat in physiological phenomena will reveal the mechanism of thermal signaling, in which the conformational changes of proteins or enhancement of enzymatic activities by a local temperature change might play a role in signal transduction. Thermal biology based on an intracellular temperature variation detected by fluorescent thermometers will make considerable contributions toward revolutionary understandings of the principles of intracellular thermal dynamics in the near future.
